# Pancreaticoduodenectomy for type I choledochal cyst in an elderly patient: a case report

**DOI:** 10.1093/jscr/rjaf477

**Published:** 2025-07-07

**Authors:** Julija Michniova, Audrius Šileikis

**Affiliations:** Faculty of Medicine, Vilnius University, M. K. Čiurlionio g. 21, LT-03101 Vilnius, Lithuania; Faculty of Medicine, Institute of Clinical Medicine, Clinic of Gastroenterology, Nephro-Urology and Surgery, Vilnius University, M. K. Čiurlionio g. 21, LT-03101 Vilnius, Lithuania

**Keywords:** choledochal cyst, congenital, elderly patient, risk of malignancy, pancreaticoduodenectomy

## Abstract

Choledochal cyst is a rare malformation of the bile ducts with high risk of malignancy. For type I cysts surgery is the mainstay of treatment. Biliary cysts are considered congenital and mostly diagnosed in children; however, the prevalence and management in adult population is poorly defined. A 77-year-old patient who presented with severe right upper quadrant pain was diagnosed with type IA choledochal cyst. Due to pancreatic head involvement in the lesion, pancreaticoduodenectomy was performed. This case emphasizes a great research gap in the understanding of choledochal malformations, especially in adults.

## Introduction

Choledochal cyst is an uncommon malformation of the intrahepatic and/or extrahepatic bile ducts with high risk of malignancy [1]. It is considered predominantly congenital and more common in children. However, the number of adults diagnosed with choledochal cysts is rising, and true prevalence is unclear. As etiopathogenesis and prognosis may differ among age groups, the optimal treatment in adult population remains poorly defined [2, 3]. Traditionally, for type I malformations, which have high malignancy rates, surgery is recommended [1]. It is, however, controversial for the elderly, who are at higher surgical risk. Type I cysts are usually managed by complete excision with either hepaticojejunostomy or hepaticoduodenostomy [4]. Pancreaticoduodenal resection may be indicated in case of extensive pancreatic head involvement, although it is extremely rare.

The case we report shows an unusually late onset of symptoms in a 77-year-old woman with type IA choledochal cyst treated with pancreaticoduodenectomy.

## Case report

A 77-year-old woman was initially referred to our department in November 2024 with the chief complaint of severe right upper quadrant pain. She was transferred from another center, as biliary cyst had been identified in magnetic resonance imaging (MRI). The patient reported that the pain had started a month earlier and had progressively worsened in the past week. On examination, she had a soft abdomen and a body mass index of 20.58. There was a history of non-specific recurrent abdominal pain since 2023. She was diagnosed with cholelithiasis the same year; however, no record of the biliary anomaly was made. Her other medical history included hypertension and hypercholesterolemia.

**Figure 1:**
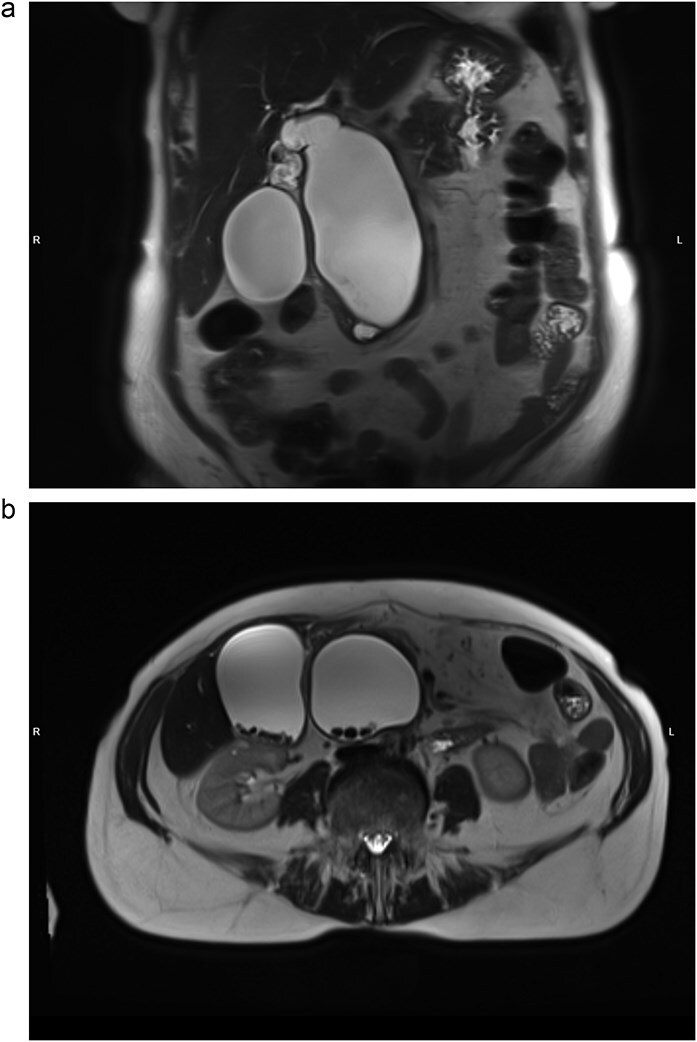
MRI, T2-weighted scan. Todani type IA biliary cyst. (a) Coronal slice: cystic dilation of CBD, diffusely dilated biliary tree; (b) axial slice: multiple CBD and gallbladder stones.

At the time of admission, laboratory findings included alkaline phosphatase of 181 U/L (normal: 40–150 U/L), GGT of 240 U/L (normal: ≤36 U/L), lipase of 318 U/L (normal: 8–78 U/L). MRI of the abdomen showed cystic dilation of the common bile duct (CBD) reaching 13 × 6 × 5 cm with infiltration of pancreatic head and multiple intraluminal fine stones (Fig. 1). Intrahepatic bile ducts, the common hepatic duct (CHD), the cystic duct and the gallbladder were also dilated, the latter contained numerous gallstones. Pancreatic head was pressed and slightly dislocated forward and downward. The bile duct cyst was subsequently classified as Todani type IA choledochal cyst.

Due to choledocholithiasis, an endoscopic retrograde cholangiopancreatography with stone extraction was performed. The procedure and postoperative course were uneventful. Considering the high risk of malignancy in choledochal cysts, complete surgical excision was indicated. A pancreaticoduodenectomy was planned owing to involvement of pancreatic head.

**Figure 2:**
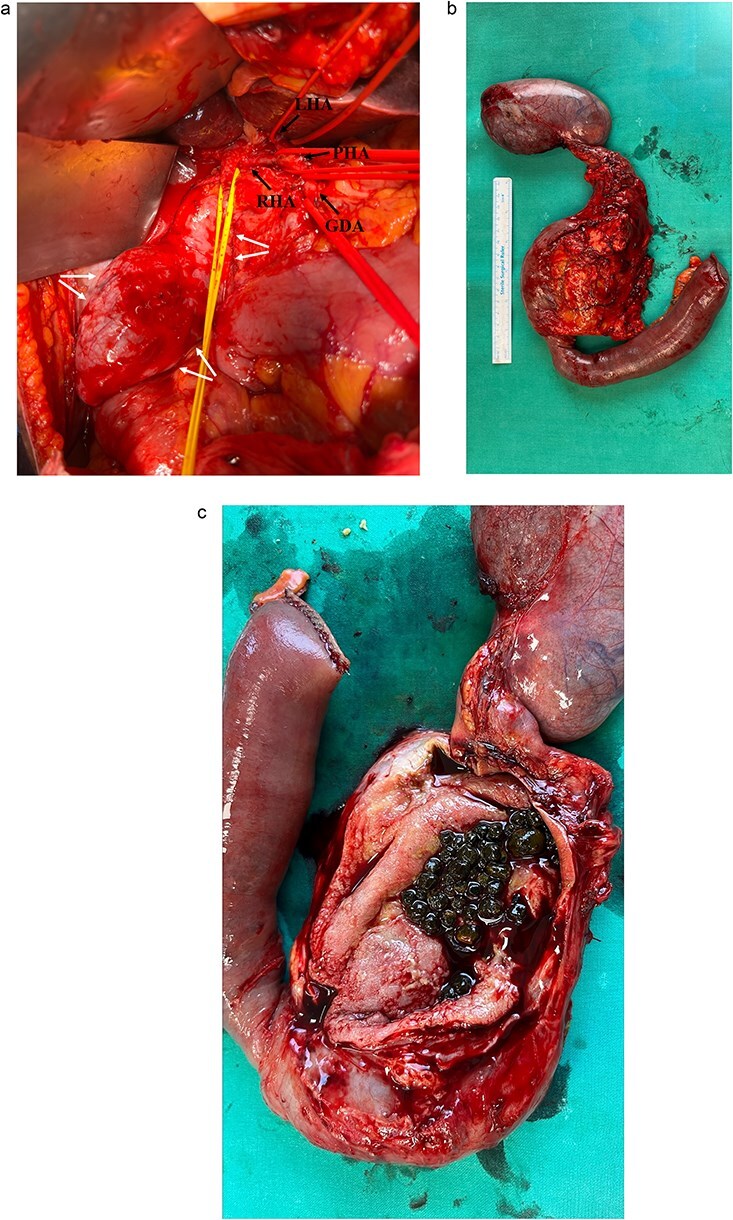
(a) Intraoperative view during dissection of the hepatoduodenal ligament showing common hepatic artery branches (single arrows), CHD (central tie) and CBD cyst (double arrows); (b) resected biliary cyst with the pancreatic head, duodenum, proximal part of jejunum and gallbladder; (c) dissected cyst containing multiple gallstones.

In January 2025 the patient was hospitalized repeatedly, and a pylorus-preserving pancreaticoduodenectomy was performed. Intraoperative aspects are shown in Fig. 2. No complications occurred during the surgery.

During the postoperative course the patient developed acute pancreatitis and a biochemical pancreatic leak. Due to delayed gastric emptying, alongside oral feeding, she was provided with enteric nutrition via nasogastric tube and parenteral nutrition for 12 and 20 days accordingly. On day 30 peripancreatic abscess was drained under local anesthesia. All resolved before the patient was discharged from the hospital on postoperative day 40.

The final pathologic examination revealed no malignant transformation, but inflammatory and ulcerous changes in the CBD, chronic cholecystitis and reactive lymphadenopathy.

## Discussion

Choledochal cysts remain of unknown origin. Most of them are linked to an abnormal pancreaticobiliary junction (APBJ), present in ⁓70% of patients [5, 6]. Historically, choledochal malformations are considered congenital. However, acquired cyst formation has also been documented in both children and adults [7, 8]. The most widely used classification, introduced by Alonso-Lej and modified by Todani [9, 10], distinguishes six types of biliary cysts according to their localization and form as well as presence of APBJ [11]. Type I cysts, characterized by dilation of the common bile duct, are further subdivided into types IA, IB, IC, and ID.

Clinically adults usually present with nonspecific abdominal pain or jaundice, yet asymptomatic disease is not uncommon [12]. What determines the timing of first symptoms is enigmatic. There has been noticed an association between clinical manifestation and the size of the lesion in prenatally diagnosed infants [13, 14]. However, evidence is limited due to small sample size. In our case, it is surprising that the patient had no symptoms until 77 years of age. Such late presentation could be indicative of unique pathogenesis and prognosis.

Management of biliary cysts in adult population is poorly defined. As the risk of developing cancer is markedly higher in adults and reported to increase with each decade [3, 6, 12], surgery remains the cornerstone of treatment, with better prognosis after complete rather than partial resection [1]. In elderly patients a conservative treatment should be considered because of increased surgical risk. Yet our patient, despite older age, had no significant comorbidities. Deferring surgery until disease progression could carry higher risk of perioperative complications and long-term morbidity due to even older age, possible novel coexisting illnesses as well as developed malignant changes.

Despite major post-surgical morbidity and long-term hospitalization, the patient’s state was good upon release. Left untreated, however, choledochal cysts could develop severe complications, the first and foremost being malignancy. The disease might also facilitate choledocholithiasis, recurrent cholangitis, cholecystitis, liver dysfunction or pancreatitis [15]. Even though conservative treatment is an option for asymptomatic elderly patients, our patient was admitted with severe pain, biliary obstruction and acute pancreatitis. Surgery was the only way for durable reduction of the symptoms and prevention of multiple complications.

Since malignancy is the main concern in choledochal malformations, its risk factors should be studied. We propose to investigate the association between the size of the lesion and risk of malignant changes, and offer data presented in the case. A novel prognostic model could help avoid intervention in those with high surgical risk.

Our case stresses a research gap in the understanding and management of choledochal cysts. For now, surgery is the optimal treatment; however, risks in the elderly should be carefully weighed on case-by-case basis.

## Data Availability

The data underlying this article will be shared on reasonable request to the corresponding author.
